# Assessing cardiovascular changes in Iraqi women with hypothyroidism

**DOI:** 10.25122/jml-2022-0220

**Published:** 2023-04

**Authors:** Suaad Muhssen Ghazi, Abdulkareem Ali Salman, Abdulmahdi Abdulameer Jawad

**Affiliations:** 1 Physiology and Medical Physics Department, College of Medicine, Al-Mustansiriyah University, Baghdad, Iraq; 2 College of Medicine, Al-Mustansiriyah University, Baghdad, Iraq; 3 National Diabetic Centre, College of Medicine, Al-Mustansiriyah University, Baghdad, Iraq

**Keywords:** echocardiography, hypothyroidism, lipid profile, ECG

## Abstract

This study aimed to investigate the cardiovascular changes associated with hypothyroidism, a topic that has received significant research attention. Although only a limited number of studies have been conducted in Iraq to evaluate cardiac parameters in patients with hypothyroidism, it is widely recognized that hypothyroidism can lead to reversible cardiac dysfunction in humans. The study enrolled 100 subjects, of which 50 were diagnosed with hypothyroidism and 50 without hypothyroidism. Medical history and body mass index (BMI) were recorded for each patient, and lipid profile, thyroid function tests, ECG, and echocardiogram results were obtained. The results revealed significant differences in the thyroid functions of hypothyroid patients compared to healthy controls, except for HDL-C, which did not show any significant difference. Hypothyroid patients had higher triglyceride and total cholesterol levels and lower HDL-C, while LDL, LDL-C, VLDL, and VLDL-C were within normal range. Patients with hypothyroidism showed a higher prevalence of ECG and echocardiogram abnormalities, including diastolic dysfunction and pericardial effusion, compared to the control subjects. Our findings suggest that hypothyroidism can affect the cardiovascular system, with the degree of impact depending on TSH elevation.

## INTRODUCTION

Hypothyroidism, a common functional disorder of the thyroid gland, is characterized by a reduction in the production of thyroid hormone or a decrease in thyroid hormone activity at the tissue level[1]. This condition affects a significant percentage of the population and accounts for over 99.5% of thyroid gland failure cases [2]. Hypothyroidism is characterized by increased serum thyrotropin (TSH) levels and low free thyroxine (T4) levels [3], and in rare cases, it may be caused by pituitary or hypothalamic dysfunction, which is known as central hypothyroidism.

Thyroid dysfunction has been linked to a range of cardiovascular issues, including an increased risk of cardiovascular morbidity and mortality in hypothyroid patients. There is a wide spectrum of dysfunction, from functional systolic/diastolic dysfunction to overt failure and coronary artery disease [4]. In hypothyroidism, the pattern of cardiac abnormalities is consistent across both subclinical and overt cases, indicating that even a slight thyroid hormone deficiency can impact the cardiovascular system. Timely detection and treatment of clinical or subclinical thyroid disorders are crucial in preventing potential cardiovascular damage [5,6].

The thyroid hormones play a crucial role in regulating the cardiovascular (CVS) system and the heart, and hypothyroidism can cause a decrease in myocardial contractility, pericardial effusion, increased left ventricular mass, and prolonged duration of contraction and relaxation. Patients suffering from overt hypothyroidism also suffer from bradycardia, decreased ventricular filling, and decreased cardiac contractility, leading to dilated cardiomyopathy and cardiac output. Patients with subclinical hypothyroidism have also been associated with systolic and diastolic cardiac dysfunction [7,8]. The ejection fraction and cardiac reserve are only slightly diminished [9].

Various clinical studies have reported that subclinical hypothyroidism is associated with changes in several cardiac parameters, including the E/A ratio, which reflects the ratio of peak blood flow induced by left ventricular relaxation in early diastole (the E wave) to peak blood flow that is caused by an atrial contraction in late diastole (the A wave)[9,10]. Electrocardiographic abnormalities such as sinus bradycardia, PR interval extension, low voltage complex, ST segment modification, flattened or inverted T waves, RBBB LBBB, and, in rare cases, complete heart block (CHB) have been observed in hypothyroidism. In echocardiography, the presence of pericardial effusion, wall motion abnormalities, and diastolic and systolic dysfunction are all signs of hypothyroidism [11].

This study aimed to evaluate the cardiac manifestations in patients with hypothyroidism by examining electrocardiogram (ECG) alterations and echocardiography findings. Additionally, we aimed to investigate the influence of obesity and dyslipidemia on these cardiac changes in hypothyroid patients.

## Material and Methods

This cross-sectional study involved 100 female participants divided into two groups: 50 patients with hypothyroidism already under thyroxine treatment and another 50 healthy control subjects who underwent echocardiography at Al-Yarmouk Teaching Hospital. Medical history and general health assessments, including BMI measurements, were recorded for each patient. In addition, laboratory investigations, including lipid profile, thyroid function test, ECG, and echocardiography findings, were performed for each participant. The lipid profile tests used in this study were total cholesterol (TC) (mg/dl), triglyceride (TG) (mg/dl), high-density lipoproteins-cholesterol (HDL-C) (mg/dl), low-density lipoproteins-cholesterol (LDL-C) (mg/dl), and very low-density lipoproteins-cholesterol VLDL-C (mg/dl). The diagnosis of hypothyroidism was confirmed by clinical evaluation and serum TSH, T4, and T3 levels, and patients with known cardiac disease, chronic obstructive pulmonary disease (COPD), or severe anemia (pernicious) were excluded. Echocardiography was performed using a 3.5 MHz transducer on a Philips (CX 50 ultrasound system) USA 2009 machine for at least 15-20 minutes. The LVEF was determined to measure systolic function and an LVEF of 50% or less indicated left ventricular systolic dysfunction. According to the guidelines set by the American Society for Echocardiography, specific measures should be obtained during echocardiography to ensure the accuracy and consistency of the results [12]. The entire process was performed under the supervision of a qualified cardiologist.

Furthermore, we evaluated diastolic function by analyzing the peak velocity blood flow during early diastole due to left ventricular relaxation (the E wave) and the peak velocity flow in late diastole caused by atrial contraction (the A wave). We calculated E/A ratios using these values, with diastolic dysfunction defined as an E/A ratio of 0.1. A ratio below 0.1 represents a reversed wave or dysfunction. The normal ranges were: T3=0.9 – 2.3 nmol/L, T4=60-120 nmol/L, TSH=0.25 – 5 µUI/L, TC <200 mg/dL, TG<150 mg/dL, HDL-C > 60 mg/dL, LDL-C <130 mg/dL, VLDL-C <30 mg/dL. The statistical analysis was performed using the IBM SPSS Statistics version 25 software. The data were presented as mean ± standard deviation (SD) or frequency (%). The independent samples t-test was used to compare continuous variables between the two groups. A p-value less than or equal to 0.05 was considered statistically significant.

## Results

The mean age of patients with hypothyroidism was 49.64 ± 11.30, while the healthy control subjects had a mean age of 51.94 ± 8.08, and there was no significant difference between the two age groups. However, the BMI of patients with hypothyroidism (32.78 ± 6.87) was significantly higher (p-value 0.0359) compared to the control group (29.94 ± 6.47). The prevalence of hypertension was not significantly different between the hypothyroidism patients (24%) and the control group (12%), with a p-value of 0.09886. The results of laboratory tests and lipid profile comparisons between the healthy control group and patients with hypothyroidism are shown in Table 1.

**Table 1:** Thyroid function and lipid profile among study participants

Laboratory tests	Hypothyroidism patients	Control	P-value
**T3(nmol/L)**	1.39 ± 0.15	2.25 ± 0.166	0.04332*
**T4 (nmol/L)**	58.28 ± 4.35	68.38 ± 4.38	<0.00001*
**TSH (µIU/L)**	3.16 ± 0.63	2.43 ± 0.36	0.00012*
**Cholesterol (mg/dl)**	206.87 ±22.72	191.18 ± 10.164	0.00041*
**Triglyceride (mg/dl)**	173.87 ± 16.73	145.88 ± 13.99	0.04161*
**HDL-C (mg/dl)**	43.41 ± 8.58	65.67 ± 5.37	0.11700 NS
**LDL-C (mg/dl)**	120.65 ± 14.30	112.12 ± 17.42	0.00854*
**VLDL-C (mg/dl)**	24.75 ± 5.56	20.86 ± 5.29	0.00052*

* – Statistical significance was defined as a p-value of 0.05 or less; NS: – Non-Significant.

There was a highly significant difference between the thyroid function levels of patients with hypothyroidism and the healthy control group. Specifically, T3 and T4 levels were lower in patients with hypothyroidism compared to the control group, while TSH levels were higher in patients with hypothyroidism. Additionally, all lipid profile tests of patients with hypothyroidism significantly differed from those of the healthy control group. TC and TG levels in patients with hypothyroidism were higher than the normal range of the control group, while the HDL-C level was lower than normal in patients with hypothyroidism. Finally, the LDL-C and VLDL-C levels of patients with hypothyroidism were higher than the control group but still within the normal range. The ECG findings comparing hypothyroidism patients and healthy controls are presented in Table 2.

**Table 2:** Electrocardiogram findings in the study groups.

Electrocardiogram finding	Hypothyroidism	Control	P-value
**Arrhythmia**	(11) 22%	(3) 6%	<0.1 NS
**Bradycardia**	(13) 26%	(5) 10%	<0.1 NS
**Low Voltage**	(16) 32%	(7) 14%	<0.1 NS

Electrocardiogram and echocardiogram findings are presented in Tables 2 and 3. Table 2 shows no significant difference in the prevalence of arrhythmia, bradycardia, and low voltage between patients with hypothyroidism and the control group. Table 3 displays the echocardiogram values for patients with hypothyroidism and control group. The echocardiogram values of the patients with hypothyroidism were slightly higher than those of the control group. There were no significant differences in the interventricular septum (IVS) and left atrium (LA) between the two groups. However, pericardial effusion was significantly higher in patients with hypothyroidism compared to control subjects. Additionally, the reversed E/A ratio was highly significant in patients with hypothyroidism compared to control individuals.

**Table 3:** Echocardiogram findings of patients with hypothyroidism and the control group.

Echocardiogram finding	Hypothyroidism Patients	Control	P-value
**IVS (mm)**	11.75 ± 1.87	10.54 ± 3.33	0.1542 NS
**L.A. size (mm)**	31.48 ± 8.36	30.82 ± 6.66	0.6634 NS
**EF (%)**	63.04 ± 6.63	61.92 ± 5.04	0.3457 NS
**E/A**	Reversed: (41) 82%	Reversed: (6) 12%	<0.0001*
**Pericardial effusion**	(2) 4%	(0) 0%	0.01*

* – Statistical significance was defined as a p-value of 0.05 or less; NS: – Non-Significant.

## Discussion

Cardiovascular consequences of long-term hypothyroidism are significant if not adequately recognized and treated early. Echocardiography, a non-invasive technique, may be very useful in identifying heart disease and monitoring treatment efficacy [13]. In this study, we assessed the heart function of female patients with hypothyroidism using ECG and echocardiogram and compared their results with healthy control subjects. The thyroid function test result showed lower levels of T3 and T4 and higher levels of TSH in patients with hypothyroidism than in the healthy control group. Our study revealed that patients with hypothyroidism had a higher BMI than controls, commonly seen in thyroid dysfunction.

In our study, obesity was observed in 12 (24%) patients with hypothyroidism. This finding is consistent with Stiefelhagen et al., who reported weight gain and dry skin in 67.5% and 62.5% of hypothyroid patients, respectively. Additionally, they found bradycardia in 40% and hypertension above 140/90 mmHg in 22.5% of hypothyroid patients [14]. An elevated lipid profile was observed in all female hypothyroid patients in our study, corroborating the findings of Stiefelhagen et al. [14]. Cardiomegaly was detected in 7.5% of hypothyroid patients, while decreased heart sounds were identified in 25%, suggesting a potential pericardial effusion. They discovered that total cholesterol increased by 15%, LDL increased by 32%, VLDL increased by 75%, triglycerides increased by 100%, and HDL decreased by. (82.5% ) [14,15].

The ECG results in our study show a higher percentage of patients with arrhythmia, bradycardia, and low voltage tests among patients with hypothyroidism than in the control group. Specifically, 11 out of 50 female hypothyroid patients had arrhythmia, 13 had bradycardia, and 16 had low-voltage ECG results. These findings align with Nikoo et al., who reported sinus tachycardia, QT prolongation, and ventricular tachycardia could occur in these patients [16]. Kishan U. and Gopala Krishna J. [17] found that 40% of the hypothyroid cases had bradycardia. 35% of the cases had low voltage complexes, and 32.5% had normal ECHO reports. 27.5% of the cases had pericardial effusion. The same proportion of cases had diastolic dysfunction. However, severe cases were rare, and only two patients exhibited IVS thickness. Stiefelhagen et al. reported abnormal ECG results in 30% of hypothyroid patients, with low voltage ECGs observed in 35% of those with aberrant results. Sinus bradycardia was the most common finding, affecting 40% of patients.

In our study, left ventricular diastolic dysfunction (LVDD) was found in 41 (82%) hypothyroid patients with a reversed E/A ratio, compared to only 6 (12%) in the control group. Our LVDD findings are consistent with previous research indicating an increased prevalence of diastolic and systolic dysfunction in hypothyroid patients, even in cases of subclinical or mild hypothyroidism [9,10,15]. The reversal of the E/A ratio, where the 'A' velocity exceeds the 'E' velocity, is a widely recognized indicator of diastolic dysfunction. This condition occurs when the left ventricular wall becomes rigid, impeding proper filling and leading to diastolic heart failure, which may develop if hypertension is left untreated for an extended period. The late phase (A) depends on atrial contraction and is absent in individuals with atrial fibrillation due to a lack of robust atrial contraction, resulting in a significantly elevated E/A ratio [18]. Hadžović-Džuvo et al. reported that the average E/A ratio, a diastolic function parameter, was lower in the hypothyroid group compared to the hyperthyroid and euthyroid groups [9, 19]. Hypothyroidism may impact the cardiovascular system depending on the severity and duration of the TSH escalation. A larger sample size is required to confirm our results, which show increased cardiovascular system involvement as TSH levels rise. Diastolic dysfunction, low voltage ECG, and abnormalities were most prevalent in ECHO and ECG findings, likely due to obesity and hypertension in these patients. According to the findings of this study, we recommended that hypothyroidism patients should undergo ECHO and ECG tests examination routinely.

## Conclusion

Our study found that age does not significantly influence the development of hypothyroidism. Hypothyroid patients were more likely to be obese compared to control females. However, we did not observe a correlation between hypertension and hypothyroidism. Hypothyroid patients exhibited substantially lower T3 and T4 levels and significantly higher TSH levels than healthy control subjects. Cholesterol and triglyceride levels were elevated in hypothyroid patients compared to controls, while HDL-C levels were lower than normal. LDL-C and VLDL-C values were higher than those in the control group but remained within the normal range. The electrocardiogram results for arrhythmia, bradycardia, and low voltage showed no significant differences between hypothyroid patients and healthy controls.

Similarly, echocardiogram measurements of the interventricular septum (IVS) and left atrium (L.A.) did not reveal significant differences between the two groups. However, the echocardiogram findings in hypothyroid patients were slightly higher than those in the control group. The prevalence of pericardial effusion was notably greater in female hypothyroid patients compared to healthy controls. Patients with hypothyroidism had a reversed E/A ratio that was statistically significant compared to healthy controls.
